# Hypophosphites in Phthisis Pulmonalis

**Published:** 1882-11

**Authors:** 


### Hypophosphites in Phthisis Pulmonalis.

Thorowgood claims a very high value in this affection for the hypophosphites, having seen better results from them than from cod oil and iron. In caseous and scrofulous pneumonia they often act like a charm. He has always found them useful and never harmful, and urges a more extensive employment of them. In using, first see that no renal or hepatic complication exists, and then test the remedy, seeing that it ignites readily when heated. Give in water or syrup. A little carb. soda may occasionally be advantageously added.—*Brit. Med. Journal.*

